# Risk Factors for Fatigue in Adults Receiving Maintenance Hemodialysis Who Have Chronic Pain: A Secondary Analysis of the HOPE Consortium Trial

**DOI:** 10.1016/j.xkme.2025.101221

**Published:** 2025-12-15

**Authors:** Mary F. Hannan, Michael J. Fischer, Jesse Hsu, Alana D. Steffen, Feiyi Sun, Kerri L. Cavanaugh, Laura M. Dember, John Farrar, Manisha Jhamb, Paul L. Kimmel, Mark B. Lockwood, Sagar U. Nigwekar, Rebecca Schmidt, Jennifer L. Steel, Mark Unruh, Ardith Z. Doorenbos

**Affiliations:** 1 Department of Biobehavioral Nursing Science, University of Illinois Chicago, College of Nursing, Chicago, IL; 2 Department of Medicine, University of Illinois Chicago, Chicago, IL; 3 Medical Service, Jesse Brown VA Medical Center, Chicago, IL; Center of Innovation for Complex Chronic Healthcare, Edward Hines Jr. VA Hospital, Hines, IL; 4 Department of Biostatistics, Epidemiology and Informatics, Center for Clinical Epidemiology and Biostatistics, University of Pennsylvania Perelman School of Medicine, Philadelphia, PA; 5 Department of Population Health Nursing Science, University of Illinois Chicago, College Nursing, Chicago, IL; 6 Division of Nephrology and Hypertension, Department of Medicine, Vanderbilt University Medical Center, Nashville, TN; 7 Nashville VA Medical Center, VA Tennessee Valley Healthcare System, Nashville, TN; 8 Renal, Electrolyte and Hypertension Division, Department of Medicine, University of Pennsylvania Perelman School of Medicine, Philadelphia, PA; 9 Department of Biostatistics, Epidemiology and Informatics, University of Pennsylvania Perelman School of Medicine, Philadelphia, PA; 10 Center for Clinical Epidemiology and Biostatistics, University of Pennsylvania Perelman School of Medicine, Philadelphia, PA; 11 Renal-Electrolyte Division, Department of Medicine, University of Pittsburgh, Pittsburgh, PA; 12 Department of Medicine, George Washington University, Washington DC; 13 Division of Nephrology, Department of Medicine, Massachusetts General Hospital, Boston, MA; 14 Harvard Medical School, Boston, MA; 15 Division of Nephrology, Department of Medicine, West Virginia University, Morgantown, WV; 16 Department of Surgery, Center for Excellence in Behavioral Medicine, University of Pittsburgh, Pittsburgh, PA; 17 Department of Internal Medicine, University of New Mexico, Albuquerque, NM; 18 Medicine Service, Raymond Murphy VA Medical Center, Albuquerque, NM

**Keywords:** Pain, end-stage kidney disease, kidney failure, dialysis, symptoms

## Abstract

**Rationale & Objective:**

Fatigue is commonly experienced by adults with kidney failure receiving hemodialysis and those with chronic pain, but factors associated with fatigue are not fully understood. We determined the prevalence of fatigue in a clinical trial cohort of adults receiving maintenance hemodialysis who have chronic pain and identified factors associated with fatigue.

**Study Design:**

A cross-sectional study.

**Setting & Participants:**

The baseline data from the HOPE Consortium Trial to Reduce Pain and Opioid Use in Hemodialysis (HOPE Trial). Of the 643 participants randomized in the HOPE Trial, 636 had a baseline fatigue assessment and were included in this study.

**Exposures:**

Pain, sociodemographic, biological, dialysis-related, medical comorbid condition, psychological, and behavioral factors.

**Outcome:**

Fatigue was evaluated with the patient-reported outcomes measurement information system Fatigue SF 6a and defined as a T-score of ≥ 55.

**Analytical Approach:**

Logistic regression models.

**Results:**

Seventy-three percent of participants reported fatigue (n = 463), mean age was 60.4 (12.5), 289 (45.4%) were female, and 294 (46.2%) were Black/African American. In fully adjusted models, higher pain interference and opioid use in the last 14 days were each associated with higher odds of having fatigue (odds ratio ([OR) ] 1.37; 95% CI, 1.18-1.61; OR 1.80; 95% CI, 1.03-3.21, respectively), as were greater depressive symptoms and sleep disturbance (OR 1.21; 95% CI. 1.13-1.31; OR 1.08 95% CI 1.03-1.12, respectively). Higher physical function was associated with lower odds of having fatigue (OR 0.96 95% CI 0.93-0.99).

**Limitations:**

Fatigue assessed at one point in time.

**Conclusions:**

In adults receiving maintenance hemodialysis who have chronic pain, pain interference, opioid use, depression, and sleep disturbances are associated with increased odds of fatigue, and greater physical function is associated with lower odds of fatigue. Future work is needed to evaluate longitudinal associations, underlying mechanisms, and identify interventions.

## Plain-Language Summary

Fatigue is commonly experienced by adults with kidney failure receiving hemodialysis and those with chronic pain, but factors associated with fatigue are not fully understood. Baseline data were examined from adults with kidney failure receiving hemodialysis who have chronic pain in the HOPE Trial. Fatigue was found in 73% of included participants. Higher levels of pain interference, opioid use, depressive symptoms, and sleep disturbances were each associated with higher odds of having fatigue. Higher levels of physical function were associated with lower odds of having fatigue. Future work is needed to evaluate causes of fatigue, to determine factors contributing to fatigue over time, and to identify interventions for fatigue in this population.

Fatigue is a symptom commonly experienced by people with kidney failure receiving maintenance dialysis.^1^ Importantly, adults with kidney failure rank fatigue as the most distressing symptom they experience and the symptom they most want treated.^2^, ^3^, ^4^ Fatigue is negatively associated with medical morbidity, mortality, health-related quality of life, social relationships, functional independence, and the ability to maintain employment in those with kidney failure.^5^, ^6^, ^7^, ^8^, ^9^, ^10^, ^11^, ^12^, ^13^ The cause of fatigue in kidney failure is poorly understood but is thought to be related to multiple dialysis, biological, psychological, and behavioral factors.^5^ Individuals with chronic pain also endure fatigue that greatly impacts quality of life, and the cause of fatigue is not fully understood, but for some may be secondary to opioid use.^14^^,^^15^

Less is known about fatigue for adults with kidney failure who experience chronic pain, which is concerning because chronic pain has been reported to be found in over 50% of adults with kidney failure.^16^ Both having pain and fatigue are associated with increased mortality in adults with kidney disease, but it is unclear how these symptoms are related and if there is a multiplicative (or synergistic) effect on health outcomes.^17^ It is well established that pain and fatigue are interconnected symptoms for people with numerous other chronic diseases.^14^^,^^15^^,^^18^, ^19^, ^20^ There has not yet been, per our review, an examination of fatigue in those with kidney failure receiving hemodialysis who experience chronic pain, or factors associated with fatigue in this population. This information is important to help identify potentially modifiable risk factors for fatigue, which will provide insight to design interventions to directly address or prevent fatigue for adults with kidney failure and chronic pain.

Therefore, the purpose of this study is to (1) determine the rates of fatigue in a clinical trial cohort of adults receiving maintenance hemodialysis who have chronic pain and (2) identify factors associated with fatigue. We hypothesize that pain intensity and interference will be associated with higher odds of having fatigue and that sociodemographic, biological, dialysis-related, medical comorbid condition, psychological, and behavioral factors will also be associated with fatigue.

## Methods

### Study Population

This study is a cross-sectional analysis of baseline data from the HOPE Consortium Trial to Reduce Pain and Opioid Use in Hemodialysis (HOPE Trial) (NCT04571619). Details of the design and primary results of the HOPE Trial have been previously published.^21^^,^^22^ For the purposes of this secondary data analysis, only baseline data were used. In brief, from January 22, 2021, to April 7, 2023, 643 adults with kidney failure and chronic pain receiving maintenance hemodialysis were randomized to either Pain Coping Skills Training, an intervention designed to increase self-efficacy for pain management, or usual care. The HOPE Trial enrolled participants from 16 centers, and participants received care at 103 dialysis units. Participants were enrolled who met the inclusion criteria, specifically adults over the age of 18 with chronic pain who had undergone maintenance hemodialysis for at least 90 days, were receiving dialysis 3 times per week, were English or Spanish speaking, had a Pain, Activity and Enjoyment of Life Scale (PEG) score greater than 4, and were willing to allow the research team to obtain the participants’ pharmacy opioid refill data and contact or work with the participant’s opioid prescriber. The major exclusion criteria were current substance use disorder, suicidal intent, substantial cognitive impairment, anticipated change in kidney replacement modality within 6 months, and life expectancy shorter than 6 months. Further details of the inclusion and exclusion criteria can be found in previously published articles.^21^^,^^22^

Of the 643 participants enrolled and randomized in the HOPE Trial, 636 completed the fatigue measure (patient-reported outcomes measurement information system [PROMIS] Fatigue SF 6a) at baseline and were included in this study. The HOPE Trial was institutional review board approved, and all participants provided written informed consent.^21^

### Factors

The factors of interest were pain interference (primary) and sociodemographic, biological, dialysis-related, medical comorbid condition, psychological and behavioral factors (secondary) that have potential relationships with fatigue in adults with kidney failure.^5^ Pain was assessed with the brief pain inventory (BPI) Severity and Interference subscales (higher scores signifying higher pain intensity and higher pain interference, respectively), which evaluate pain over the last 7 days. The BPI interference subscale evaluates the degree that pain interferes with an individual’s life, and it is evaluated with questions on how much pain has interfered with various aspects of an individual’s life (eg, physical activity, relationships, mood, enjoyment, and sleep).^23^ Opioid use within the last 14 days was also examined, which was determined via timeline follow back.

The sociodemographic factors examined were age, sex at birth, race and ethnicity, employment status, relationship status, and level of education, all of which were self-reported at baseline and collected from participants by trained staff using case report forms. The dialysis-related factors examined were dialysis adequacy (Kt/V) and the number of years receiving hemodialysis. The biological and medical comorbid condition factors evaluated were hemoglobin (g/dL), albumin (g/dL), urea nitrogen (BUN) (mg/dL), and history of comorbid health conditions, specifically cardiovascular diseases (coronary heart disease, congestive heart failure, atrial fibrillation, arrhythmia, stroke, peripheral vascular disease, and valvular disease), diabetes, cancer, chronic lung disease, inflammatory bowel disease, arthritis, systemic lupus erythematosus, vasculitis, sickle cell anemia, and hyperparathyroidism (represented as taking either cinacalcet or a vitamin D analog). The psychological and behavioral factors examined were level of social support (assessed by the Multidimensional Scale of Perceived Social Support [MSPSS]), anxiety (General Anxiety Disorder-7 [GAD-7]), depressive symptoms (Patient Health Questionnaire-9 [PHQ-9]), sleep disturbances or disorders (PROMIS sleep disturbance instrument), self-reported physical functioning (PROMIS physical functioning SF 6b), and substance use (smoking status, alcohol use, and marijuana use).

### Outcome

Fatigue was the outcome of interest. Fatigue was evaluated with the PROMIS Fatigue SF 6a, which evaluates the level of fatigue over the last 7 days. The PROMIS Fatigue SF 6a evaluates an individual’s overall experience of fatigue via 6 questions that examine the severity, bothersomeness, and interference of fatigue. Fatigue, along with other self-report measures, was collected by a centralized patient-reported outcomes ascertainment team. The PROMIS Fatigue 6a is scored on a 6-30 scale and then converted to a T-score (a mean of 50 with a standard deviation [SD] of 10) with higher scores representing more fatigue. We used the cutoff T-score of above 55 to classify the presence of fatigue and used cutoffs for mild fatigue (score 55-59), moderate fatigue (score 60-69), and severe fatigue (score ≥ 70), as has been done in other work in the general population and based on guidance from the HealthMeasures scoring service.^24^, ^25^, ^26^ While there is no established cutoff for a clinically significant change in PROMIS Fatigue 6a score or established T-score cutoffs for levels of fatigue severity for adults receiving maintenance hemodialysis, research using the PROMIS Fatigue Computer Adaptive Test (PROMIS-F CAT) found that a T-score ≥59 reflects clinically relevant fatigue in those receiving hemodialysis.^27^ Additionally, in other clinical populations on different short form versions of the PROMIS Fatigue measure, a score change of 3-5 has been found to be clinically meaningful.^28^, ^29^, ^30^

### Statistical Analysis

Means and standard deviations were used to summarize continuous variables. Categorical variables were summarized using counts and percentages. Univariate logistic regression models were conducted to examine the associations between the hypothesized factors and the presence of fatigue. To identify factors associated with fatigue, multivariable analyses were conducted through hierarchical incorporation of factors showing univariate associations with significance levels of *P* < 0.05 to provide adjusted estimates of association with fatigue. Significant subgroups of secondary factors were added according to content domain: (1) sociodemographic, (2) biological, medical comorbid condition, and dialysis-related, and (3) psychological and behavioral factors. Multicollinearity was evaluated for in the hierarchical models, and there was no evidence of multicollinearity other than in expected relationships (ie, pain severity and interference). As a sensitivity analysis, we also examined the relationship between the factors and fatigue in linear regression models. Missing data were excluded from the analysis (missing data consisted of the following (Table S1): BPI interference: 1, BPI severity: 3, multidimensional scale of perceived social support: 10, general anxiety disorder-7: 3, patient health questionnaire-9: 14, PROMIS sleep: 7, PROMIS physical function: 1, Kt/V: 66, hemoglobin: 7, albumin: 15, urea nitrogen: 22, cardiovascular disease: 20, diabetes mellitus: 1, and history of cancer: 1). For all analyses, the overall level of significance was set to α = 0.05. Data analyses were performed using R (version 4.3.2; https://cran.r-project.org/).

## Results

**Figure 1:**
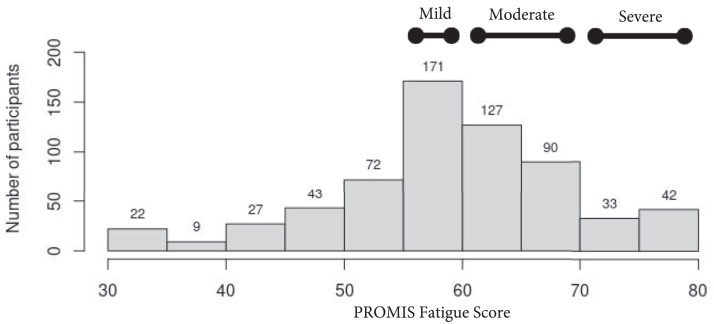
Histogram of fatigue scores.

The mean and SD age of the sample was 60.4 ± 12.5 years, 289 (45.4%) were female, and the mean ± SD pain interference rating was 6.4 ± 2.1 and pain severity rating was 5.9 ± 2.1. (Table 1) Fatigue, as indicated by a PROMIS Fatigue T-score ≥ 55, was reported by 463 participants (73%). Of the sample, 143 (22%) had mild fatigue, 245 (39%) had moderate fatigue, and 75 (12%) had severe fatigue, and the distribution of fatigue scores was normally distributed (Fig 1). Those with fatigue had higher pain interference and pain severity scores, higher opioid use in the last 14 days, higher anxiety scores, worse depressive symptoms, and higher sleep disturbance. In contrast, those with fatigue had lower social support scores and lower physical functioning. (Table 1).

**Table 1:** Participant Characteristics by Presence of Fatigue^a^

Characteristic	Overall N = 636	No Fatigue (n = 173)	Fatigue (n = 463)	*P*
BPI interference^b^	6.4 (2.1)	5.0 (2.2)	6.9 (1.8)	<0.001
BPI severity^b^	5.9 (2.1)	5.0 (2.2)	6.2 (2.0)	<0.001
Opioid use in the last 14 days	162 (25.5%)	29 (16.8%)	133 (28.7%)	0.002
Age (y)	60.4 (12.5)	60.6 (13.2)	60.3 (12.2)	0.98
Female (sex at birth)	289 (45.4%)	77 (44.5%)	212 (45.8%)	0.79
Race and ethnicity				0.86
Hispanic	118 (18.6%)	32 (18.5%)	86 (18.6%)	
Non-Hispanic Black	294 (46.2%)	84 (48.6%)	210 (45.4%)	
Non-Hispanic White	173 (27.2%)	43 (24.9%)	130 (28.1%)	
Other^c^	51 (8.0%)	14 (8.1%)	37 (8.0%)	
Currently not employed	584 (91.8%)	158 (91.3%)	426 (92.0%)	0.78
Currently married/domestic partner	197 (31.0%)	63 (36.4%)	134 (28.9%)	0.19
Less than high school	137 (21.5%)	35 (20.2%)	102 (22.0%)	0.95
Years on dialysis				0.58
<1 y	142 (22.3%)	35 (20.2%)	107 (23.1%)	
1-5 y	295 (46.4%)	79 (45.7%)	216 (46.7%)	
>5 y	199 (31.3%)	59 (34.1%)	140 (30.2%)	
Kt/V^b^	1.6 (0.3)	1.6 (0.3)	1.6 (0.3)	0.09
Hemoglobin (g/dL)^b^	10.9 (1.7)	11.0 (1.9)	10.8 (1.6)	0.99
Albumin (g/dL)^b^	3.9 (0.4)	3.9 (0.4)	3.9 (0.4)	0.73
Urea Nitrogen (mg/dL)^b^	54.0 (22.5)	54.1 (20.7)	53.9 (23.1)	0.94
Cardiovascular disease^b^	386 (62.7%)	104 (61.5%)	282 (63.1%)	0.72
Diabetes mellitus^b^	379 (59.7%)	95 (55.2%)	284 (61.3%)	0.16
History of cancer^b^	105 (16.5%)	29 (16.8%)	76 (16.5%)	0.92
History of chronic lung disease^b^	75 (11.9%)	20 (11.7%)	55 (11.9%)	0.94
History of inflammatory bowel disease^b^	22 (3.5%)	7 (4.1%)	15 (3.3%)	0.60
History of arthritis^b^	341 (54.9%)	91 (54.5%)	250 (55.1%)	0.90
History of systemic lupus erythematosus^b^	20 (3.2%)	5 (2.9%)	15 (3.2%)	0.85
History of sickle cell anemia^b^	10 (1.6%)	3 (1.8%)	7 (1.5%)	0.73
Hyperparathyroidism^d^	272 (42.8%)	76 (43.9%)	196 (42.3%)	0.72
MSPSS total score^b^	5.1 (1.5)	5.6 (1.4)	5.0 (1.5)	<0.001
GAD-7^b^	7.1 (6.0)	3.2 (4.0)	8.5 (6.0)	<0.001
PHQ-9^b^	9.2 (6.0)	4.5 (3.8)	10.9 (5.7)	<0.001
PROMIS sleep disturbance (raw)^b^	19.4 (6.6)	14.9 (6.3)	21.1 (5.9)	<0.001
PROMIS physical function (T-score)^b^	33.3 (7.6)	37.0 (8.8)	32.0 (6.6)	<0.001
Current smoker	98 (15.4%)	27 (15.6%)	71 (15.3%)	0.53
Current alcohol use	63 (9.9%)	19 (11.0%)	44 (9.5%)	0.58
Current marijuana use	99 (15.6%)	22 (12.7%)	77 (16.6%)	0.23

Abbreviations: BPI, brief pain inventory; GAD-7, general anxiety disorder-7; MSPSS, multidimensional scale of perceived social support; PHQ-9, patient health questionnaire-9; PROMIS, patient-reported outcomes measurement information system.

a Fatigue defined as PROMIS Fatigue T-score ≥ 55.

b See Table S1 for missing data count.

c Other race or ethnicity included non-Hispanic participants who are American Indian/Alaskan Native, Asian/Asian American, Native Hawaiian/other Pacific Islander, multi-race, or not reported.

d Hyperparathyroidism defined as taking either cinacalcet or a vitamin D analog.

In unadjusted models (Table 2; Column 1), pain interference, pain severity, and opioid use in the last 14 days were each associated with higher odds of having fatigue (odds ratio [OR] 1.62; 95% confidence interval [CI] 1.47-1.80; OR 1.32; 95% CI, 1.21-1.44; and OR 2.00; 95% CI, 1.30-3.18, respectively). Additionally, in unadjusted models, social support, anxiety, depression, sleep disturbance, and physical function were each associated with fatigue (Table 2; Column 1). In the fully adjusted model (Table 2; Column 4) that adjusted for the sociodemographic, biological, dialysis-related, comorbid medical conditions, psychological, and behavioral factors that were significant in unadjusted models, pain interference and opioid use in the last 14 days persisted as being associated with higher odds of having fatigue (OR 1.37; 95% CI, 1.18-1.61 and OR 1.80; 95% CI, 1.03-3.21), as did more depressive symptoms and a higher sleep disturbance score (OR 1.21; 95% CI, 1.13-1.31 and OR 1.08; 95% CI, 1.03-1.12, respectively), and higher physical function persisted as being associated with lower odds of having fatigue (adjusted model OR 0.96; 95% CI, 0.93-0.99). Although in the unadjusted model higher pain severity and higher anxiety were each associated with higher odds of having fatigue and higher social support was associated with lower odds of having fatigue, these were no longer significant in the fully adjusted model. In sensitivity analyses exploring the relationship of the factors with fatigue in ordinary least squares regressions, similar relationships were identified in the fully adjusted model. Of particular note, each 1-point increase in pain interference score was associated with a 0.82 increase in fatigue score (95% CI, 0.42-1.23), each 1-point increase in depressive symptom score was associated with 0.48 increase in fatigue score (95% CI, 0.33-0.63) (Table S2). Conversely, opioid use within the last 14 days was no longer significantly associated with fatigue scores in the fully adjusted model (beta 0.89; 95% CI, −0.49 to 2.26) but anxiety remained significantly associated with fatigue in the fully adjusted model (beta 0.20; 95% CI, 0.06-0.34) (Table S2).

**Table 2:** Relationship Between Factors and Fatigue

Factors	Unadjusted Models OR (95% CI) (n = 636)^a^	Model 1 OR (95% CI) (n = 623)^b^	Model 2 OR (95% CI) (n = 623)^c^	Model 3 OR (95% CI) (n = 600)^d^
Pain				
BPI interference	1.62 (1.47-1.80)	1.66 (1.47-1.89)	1.66 (1.47-1.89)	1.37 (1.18-1.61)
BPI severity	1.32 (1.21-1.44)	0.96 (0.85-1.08)	0.96 (0.85-1.08)	0.93 (0.80-1.07)
Opioid use in the last 14 days				
No	1.00	1.00	1.00	1.00
Yes	2.00 (1.30-3.18)	1.84 (1.15-3.04)	1.84 (1.15-3.04)	1.80 (1.03-3.21**)**
Sociodemographic factors				
Age (y)	1.00 (0.98-1.01)			
Sex				
Male	1.00			
Female	1.05 (0.74-1.50)			
Race and/or ethnicity				
Non-Hispanic White	1.00			
Non-Hispanic Black	0.83 (0.54-1.26)			
Hispanic	0.89 (0.52-1.52)			
Other	0.87 (0.44-1.81)			
Current employment status				
Currently employed	1.00			
Not employed	1.09 (0.57-2.01)			
Current relationship status				
Married/domestic partner	1.00			
Widowed/divorced/separated	1.39 (0.92-2.13)			
Never married	1.42 (0.92-2.20)			
Education				
Less than high school	1.00			
High school degree	0.91 (0.57-1.45)			
Associate's or technical degree	0.85 (0.48-1.50)			
College degree, doctoral, or postgraduate education	0.91 (0.53-1.58)			
Biological, dialysis-related, and medical comorbid condition factors				
Dialysis vintage				
<1 y	1.00			
1-5 y	0.89 (0.56-1.41)			
>5 y	0.78 (0.47-1.26)			
Kt/V	0.73 (0.41-1.31)			
Hemoglobin (g/dL)	0.93 (0.84-1.04)			
Albumin (g/dL)	0.96 (0.61-1.50)			
Urea nitrogen (mg/dL)	1.00 (0.99-1.01)			
History of any cardiovascular disease				
No	1.00			
Yes	1.07 (0.74-1.54)			
History of diabetes mellitus				
No	1.00			
Yes	1.29 (0.90-1.83)			
History of cancer diagnosis				
No	1.00			
Yes	0.98 (0.62-1.58)			
History of chronic lung disease				
No	1.00			
Yes	1.02 (0.60-1.8)			
History of inflammatory bowel disease				
No	1.00			
Yes	0.79 (0.33-2.09)			
History of arthritis				
No	1.00			
Yes	1.02 (0.72-1.46)			
History of systemic lupus erythematosus				
No	1.00			
Yes	1.1 (0.42-3.44)			
History of sickle cell anemia				
No	1.00			
Yes	0.86 (0.24-4.03)			
Hyperparathyroidism				
No	1.00			
Yes	0.94 (0.66-1.33)			
Psychological and behavioral factors				
Social Support (MSPSS total score)	0.74 (0.64-0.84)			0.93 (0.79-1.10)
GAD-7 (anxiety)	1.24 (1.19-1.30)			1.03 (0.97-1.10)
PHQ-9 (depression)	1.36 (1.29-1.44)			1.21 (1.13-1.31)
PROMIS sleep disturbance	1.18 (1.14-1.22)			1.08 (1.03-1.12)
PROMIS physical function	0.91 (0.89-0.93)			0.96 (0.93-0.99)
Smoking status				
Current smoker	1.00			
Former smoker	1.17 (0.68-1.99)			
Never smoker	0.93 (0.56-1.53)			
Alcohol use				
No	1.00			
Yes	0.85 (0.49-1.53)			
Current marijuana use				
No	1.00			
Yes	1.37 (0.84-2.33)			

*Note:* Hyperparathyroidism defined as taking either cinacalcet or vitamin D analog.

Abbreviations: BPI, brief pain inventory; GAD-7, general anxiety disorder-7; MSPSS, multidimensional scale of perceived social support; PHQ-9, patient health questionnaire-9; PROMIS, patient-reported outcomes measurement information system.

a Unadjusted Logistic regression model separately predicts fatigue with each factor.

b Model 1 adjusts for pain and sociodemographic factors that show statistically significant evidence of association with fatigue (*P* < 0.05) in the unadjusted model.

c Model 2 adjusts for pain, sociodemographic, and physiological/dialysis-related factors that show statistically significant evidence of association with fatigue (*P* < 0.05) in the unadjusted model. Model 1 and model 2 have the same outputs since no physiological/dialysis-related factors are significant.

d Model 3 adjusts for pain, sociodemographic, physiological/dialysis-related, and psychological/behavioral factors that show statistically significant evidence of association with fatigue (*P* < 0.05) in the unadjusted model.

## Discussion

We found that fatigue is extremely common in adults with kidney failure receiving maintenance hemodialysis who have chronic pain. Our report is among the first to examine fatigue specifically in adults receiving hemodialysis who have chronic pain. We found that almost 73% of the sample had fatigue, including 12% with severe fatigue. Pain interference, recent opioid use, depressive symptoms, sleep disturbance, and low physical functioning were independently associated with increased odds of fatigue. Identifying potentially modifiable factors associated with fatigue is important since fatigue is highly distressing for people with kidney failure.^2^, ^3^, ^4^ Our findings are particularly relevant since they describe fatigue in adults receiving maintenance hemodialysis who have chronic pain, a population where fatigue and factors that influence fatigue have not been fully characterized.

We found the prevalence of fatigue in a clinical trial cohort of adults receiving maintenance hemodialysis who have chronic pain was 73%, which is similar to the prevalence found in other samples of adults receiving maintenance hemodialysis.^1^ Our findings on the association between pain and fatigue are also congruent with other studies in other patient populations that have found associations between pain and fatigue, but our findings extend what is known about pain and fatigue by examining this relationship in adults receiving maintenance hemodialysis who have chronic pain. Chronic pain and fatigue are believed to be intricately linked, although the temporality of this relationship is less clear.^14^^,^^31^ The relationship between pain and fatigue is well established in older adults and people with other chronic diseases,^14^^,^^15^^,^^18^^,^^19^^,^^32^^,^^33^ and in those with kidney disease.^20^^,^^34^ The relationship between pain and fatigue has been less studied in those with kidney failure receiving hemodialysis who are being treated for chronic pain. This may be due to the complexity of factors that may potentially influence fatigue in those with kidney disease given that numerous dialysis-related, physiological, sociodemographic, and psychological, and behavioral factors have been found to be associated with fatigue in those receiving maintenance hemodialysis.^5^ Fatigue has been moderately correlated with pain in those with kidney failure, as well as in people with chronic kidney disease.^20^ Our findings add to this area of knowledge by looking specifically at adults with kidney failure receiving maintenance hemodialysis who have chronic pain and examining overall fatigue, rather than just fatigue related to the dialysis procedure itself.

The findings of this study are also consistent with what has been found about the relationship between depression and fatigue in those with kidney disease. In those with kidney failure, as well as in those with chronic kidney disease, depression and fatigue have been found to be related, specifically that adults with kidney failure and depressive symptoms had greater levels of fatigue than those without depressive symptoms and that depressive symptoms have been correlated with higher fatigue levels.^35^, ^36^, ^37^ The role of treating depression to improve fatigue in those with kidney failure is less clear^5^ but has been investigated as secondary outcome in one clinical trial focused on treating depression in adults with kidney failure receiving hemodialysis, where treatment of depression resulted in improved energy and vitality.^38^^,^^39^ This study adds to what is known about depression and fatigue by highlighting the relationship between depression and fatigue in adults receiving maintenance hemodialysis who have chronic pain.

The results of this study that subjective sleep disturbances are associated with fatigue are similar to findings from the general population^40^ and found in adults with other chronic diseases, including multiple sclerosis and cancer.^41^, ^42^, ^43^ In adults with kidney failure, poor self-reported sleep quality and sleep latency (time it takes to fall asleep) have been related to fatigue.^44^^,^^45^ This is particularly relevant because adults with kidney failure commonly have sleep disorders that are related to the fatigue.^46^ Although treating sleep disturbances has improved fatigue in other populations,^47^^,^^48^ it does not appear to be effective at improving fatigue in those with kidney failure^49^ but further work in this area is needed.

We found that self-reported lower physical function was also associated with higher fatigue. Objective measures of physical function (ie, handgrip strength, chair-rise time) have been associated with fatigue in the general population and in those with cancer,^50^^,^^51^ as have subjective measures of physical function in those with arthritis and those with chronic fatigue syndrome.^52^^,^^53^ The association of physical function and fatigue has also been found in adults with incident kidney failure, specifically that lower self-reported physical function was associated with fatigue (lower vitality scores).^44^ These findings highlight that person-centered behavioral and physical activity interventions directed at increasing physical function in adults receiving maintenance hemodialysis who have chronic pain may promote improved symptoms.

Similar to our unadjusted analysis results, other work has found higher perceived social support to be associated with lower fatigue^54^ and higher anxiety to be associated with higher fatigue in those with kidney failure.^36^^,^^37^ Interestingly, we found that the relationship of social support and anxiety with fatigue did not persist after adjustment for other factors, which suggests that anxiety and social support may be less explanatory than other predictor variables or that these factors may be in the causal pathway between other predictor variables leading to fatigue. Further research with regard to the association between social support, anxiety, and fatigue warrants further investigation.

In contrast to other research with adults with kidney failure,^11^^,^^37^^,^^44^^,^^55^, ^56^, ^57^ we did not find associations between the demographic factors of sex and age and the biological factors, such as anemia, comorbid medical conditions, and albumin level, with fatigue. The reason for our dissimilar findings may be that we looked specifically at a population of adults receiving maintenance hemodialysis with chronic pain, who may have potentially different factors that influence their fatigue, given the complex interrelationship between pain and fatigue. Additionally, the variability in the biological, medical comorbid condition, and dialysis-related factors among HOPE participants may not have been sufficient to observe a relationship in this study. Finally, the sample size did not warrant detailed exploration into subgroup analyses by demographic factors, but this investigation should be pursued with larger samples of adults with chronic pain receiving hemodialysis.

Despite our study’s strengths in its exploration of a large sample of a diverse population of adults receiving maintenance hemodialysis who have chronic pain, it has limitations. Our study was cross-sectional, so causal inferences cannot be made. The HOPE Trial did not use probability sampling methods, so the sample does not represent the entire population of adults with kidney failure and chronic pain; however, the participants came from 103 dialysis facilities in several geographic areas in the United States. Fatigue and the predictors of interest were measured at one point in time for this analysis, so may not truly reflect the fluctuations in fatigue that may be observed. Fatigue was measured at one point in time and participants were not instructed to fill out the measure describing their generalized fatigue or the fatigue they may experience during or after dialysis. Given that fatigue level may vary in relation to the dialysis schedule,^58^ the PROMIS Fatigue SF 6a and its assessment of fatigue over the last 7 days may not fully capture the variable fatigue experience of adults with kidney failure receiving hemodialysis. PROMIS Fatigue scores were evaluated based on T-score cutoffs established in the general population. We acknowledge that there are limitations to using T-score cutoffs not established in adults receiving hemodialysis. Therefore, we also examined the relationship between factors and fatigue in regression models as a sensitivity analysis, and the findings were similar. The factors included in this analysis were limited to those available in the parent study, so not all potential factors associated with fatigue were able to be examined. Although the BPI has been commonly used to assess pain in adults with kidney failure, it may not capture the full pain experience of adults with kidney failure receiving hemodialysis.^59^ We could not control for all medications that influence fatigue, although we did examine opioid use, which was associated with fatigue.

In conclusion, in this study of adults with kidney failure and chronic pain, pain interference, and several potentially modifiable psychological and behavioral factors, was associated with increased odds of having fatigue. Future work is needed investigating longitudinal relationships between pain and fatigue and interventions to address pain, psychological, and behavioral factors to potentially reduce fatigue in those with kidney failure and chronic pain.
