# Supplementary material for: Risk Factors for Fatigue in Adults Receiving Maintenance Hemodialysis Who Have Chronic Pain: A Secondary Analysis of the HOPE Consortium Trial
Source: Kidney Med. 2025 Dec 15;8(2):101221. doi: 10.1016/j.xkme.2025.101221 (PMC12856481; doi:10.1016/j.xkme.2025.101221)
Supplement: Supplementary File (PDF) — Tables S1, S2. [file mmc1.pdf]

Table S1. Missing Data Table

**Characteristic**	**Missing**
BPI Interference	1
BPI Severity	3
MSPSS total score	10
GAD-7 (anxiety)	3
PHQ-9 (depression)	14
PROMIS sleep disturbance raw score	7
PROMIS physical function T score	1
Kt/V	66
Hemoglobin (g/dL)	7
Albumin (g/dL)	15
Urea Nitrogen (mg/dL)	22
Cardiovascular disease	20
Diabetes mellitus	1
History of cancer	1

Table S2. Relationship between factors and fatigue (OLS Regression)

Factors	Unadjusted Models Beta (95% CI) (N=636)[1]	Model 1 Beta (95% CI) (N=623)[2]	Model 2 Beta (95% CI) (N=623)[3]	Model 3 Beta (95% CI) (N=600)[4]
**Pain**				
BPI Interference	**2.16 (1.83, 2.50)**	**2.11 (1.68, 2.55)**	**2.08 (1.65, 2.52)**	**0.82 (0.42, 1.23)**
BPI Severity	**1.42 (1.06, 1.77)**	0.02 (-0.42, 0.45)	0.04 (-0.39, 0.47)	-0.1 (-0.48, 0.27)
Opioid use in the last 14 days	**2.57 (0.81, 4.33)**	**1.69 (0.10, 3.27)**	**1.65 (0.05, 3.24)**	0.89 (-0.49, 2.26)
**Sociodemographic Factors**				
Age (years)	-0.05 (-0.12, 0.01)			
Sex- Female (Ref Male)	1.34 (-0.20, 2.89)			
Race and/or ethnicity				
Non-Hispanic White	-0.83 (-2.70, 1.04)			
Non-Hispanic Black	0.31 (-2.02, 2.63)			
Hispanic	-0.05 (-3.16, 3.05)			
Other				
Current employment status- not employed (Ref. employed)	1.04 (-1.78, 3.85)			
Current relationship status				
Married/partner	Ref			
Widowed/divorced/separated	**2.02 (0.14, 3.89)**	1.05 (-0.64, 2.73)	1.01 (-0.69, 2.70)	-0.08 (-1.53, 1.38)
Never married	1.25 (-0.68, 3.19)	0.47 (-1.27, 2.20)	0.3 (-1.44, 2.05)	0.27 (-1.28, 1.81)
Education				
Less than high school	Ref			
High school degree	-0.35 (-2.40, 1.69)			
Associate's or technical degree	-1.00 (-3.54, 1.53)			

Factors	Unadjusted Models Beta (95% CI) (N=636)[1]	Model 1 Beta (95% CI) (N=623)[2]	Model 2 Beta (95% CI) (N=623)[3]	Model 3 Beta (95% CI) (N=600)[4]
College degree, doctoral, or postgraduate education	-0.07 (-2.47, 2.32)			
**Dialysis-Related & Physiological Factors**				
Dialysis vintage				
<1 year	Ref			
1-5 years	-1.27 (-3.25, 0.72)			
>5 years	-0.54 (-2.67, 1.60)			
Kt/V	-1.41 (-4.00, 1.17)			
Hemoglobin (g/dL)	-0.30 (-0.77, 0.16)			
Albumin (g/dL)	0.08 (-1.90, 2.06)			
Urea Nitrogen (mg/dL)	0.02 (-0.02, 0.06)			
History of any cardiovascular disease				
No	Ref			
Yes	-0.20 (-1.81, 1.42)			
History of diabetes mellitus				
No	Ref			
Yes	0.98 (-0.59, 2.55)			
History of cancer diagnosis				
No	Ref			
Yes	-0.96 (-3.03, 1.12)			
History of chronic lung disease				
No	Ref			
Yes	1.40 (-0.99, 3.79)			
History of inflammatory bowel disease				

Factors	Unadjusted Models Beta (95% CI) (N=636)[1]	Model 1 Beta (95% CI) (N=623)[2]	Model 2 Beta (95% CI) (N=623)[3]	Model 3 Beta (95% CI) (N=600)[4]
No	Ref			
Yes	1.21 (-3.02, 5.44)			
History of arthritis				
No	Ref			
Yes	-0.31 (-1.87, 1.25)			
History of systemic lupus erythematosus				
No	Ref			
Yes	1.37 (-3.03, 5.78)			
History of sickle cell anemia				
No	Ref			
Yes	0.26 (-5.94, 6.46)			
Hyperparathyroidism				
No	Ref			
Yes	-0.14 (-1.70, 1.42)			
**Psychological & Behavioral Factors**				
Social Support (MSPSS total score)	**-1.15 (-1.65, -0.65)**			0.14 (-0.27, 0.56)
GAD-7 (anxiety)	**0.84 (0.73, 0.95)**			**0.20 (0.06, 0.34)**
PHQ-9 (depression)	**0.97 (0.86, 1.08)**			**0.48 (0.33, 0.63)**
PROMIS sleep disturbance	**0.75 (0.65, 0.85)**			**0.30 (0.19, 0.40)**
PROMIS physical function	**-0.47 (-0.56, -0.37)**			**-0.19 (-0.28, -0.10)**
Smoking status				
Current Smoker	Ref			
Former smoker	-0.22 (-2.58, 2.15)			

Factors	Unadjusted Models Beta (95% CI) (N=636)[1]	Model 1 Beta (95% CI) (N=623)[2]	Model 2 Beta (95% CI) (N=623)[3]	Model 3 Beta (95% CI) (N=600)[4]
Never smoker	-0.86 (-3.11, 1.39)			
Alcohol Use				
No	Ref			
Yes	-0.94 (-3.52, 1.64)			
Current marijuana use				
No	Ref			
Yes	**2.53 (0.41, 4.65)**			0.99 (-0.65, 2.64)

BPI, Brief Pain Inventory; GAD-7, General anxiety disorder-7; PHQ-9, Patient Health Questionnaire-9; PROMIS, Patient-Reported Outcomes Measurement Information System; MSPSS, Multidimensional Scale of Perceived Social Support; Hyperparathyroidism defined as taking either cinacalcet or vitamin D analogue

[1]Unadjusted OLS regression model separately predicts fatigue with each covariate.

[2]Model 1 adjusts for pain and sociodemographic factors that show statistically significant evidence of association with fatigue (p<0.05) in the unadjusted model.

[3]Model 2 adjusts for pain, sociodemographic, and physiological/dialysis-related factors that show statistically significant evidence of association with fatigue (p<0.05) in the unadjusted model.

[4]Model 3 adjusts for pain, sociodemographic, physiological/dialysis-related, and psychological/behavioral factors that show statistically significant evidence of association with fatigue (p<0.05) in the unadjusted model.
